# SIRT2, a direct target of miR‐212‐5p, suppresses the proliferation and metastasis of colorectal cancer cells

**DOI:** 10.1111/jcmm.15603

**Published:** 2020-07-22

**Authors:** Feng Du, Zhijun Li, Guohua Zhang, Si Shaoyan, Dejun Geng, Zhougen Tao, Kunhua Qiu, Silei Liu, Yu Zhou, Yichao Zhang, Jianwen Gu, Gang Wang, Lianyong Li, Wei Wu

**Affiliations:** ^1^ Department of Gastroenterology PLA Strategic Support Force Characteristic Medical Center Beijing China; ^2^ State Key Laboratory of Environmental Sense Organ Stress and Health of the Ministry of Environmental Protection PLA Strategic Support Force Characteristic Medical Center Beijing China; ^3^ Department of Internal Medicine The Hospital of the People's Liberation Army 63650 Corps Malan China; ^4^ Laboratory of Basic Medical Research PLA Strategic Support Force Characteristic Medical Center Beijing China; ^5^ Department of Neurological Surgery PLA Strategic Support Force Characteristic Medical Center Beijing China; ^6^ Department of Otorhinolaryngology Head and Neck Surgery PLA Strategic Support Force Characteristic Medical Center Beijing China

**Keywords:** colorectal cancer, metastasis, miR‐212‐5p, prognosis, sirtuin 2, tumorigenesis

## Abstract

The aberrant expression of human sirtuin 2 (SIRT2) has been detected in various types of cancer; however, the biological roles, underlying mechanisms and clinical significance of SIRT2 dysregulation in human colorectal cancer (CRC) remain unclear. The results of this study demonstrate that compared with paired normal tissues, SIRT2 expression is significantly decreased in CRC tissues. SIRT2 loss has been correlated with clinicopathological characteristics, including distant metastasis, lymph node metastasis and American Joint Committee on Cancer (AJCC) stage; this loss serves as an independent factor that indicates a poor prognosis for patients with CRC. Further gain‐ and loss‐of‐function analyses have demonstrated that SIRT2 suppresses CRC cell proliferation and metastasis both in vivo and in vitro. Mechanistically, miR‐212‐5p was identified to directly target the SIRT2 3′‐untranslated region (3′‐UTR), leading to SIRT2 down‐regulation. The ectopic expression of SIRT2 reverses the effect of miR‐212‐5p overexpression on CRC cell colony formation, invasion, migration and proliferation. Clinically, an inverse correlation was found between miR‐212‐5p and SIRT2 expression. High miR‐212‐5p expression has been found to result in a poor prognosis and aggressive clinicopathological characteristics in patients with CRC. Taken together, these results suggest that SIRT2, targeted by miR‐212‐5p, acts as a tumour suppressor in CRC and that the miR‐212‐5p/SIRT2 axis is a promising prognostic factor and potential therapeutic target in CRC.

## 1 INTRODUCTION

Worldwide, it has been found that colorectal cancer (CRC) results in the third highest number of malignancies and is the fourth leading cause of cancer‐related mortality.^1^ Despite the development of systemic treatments for patients with CRC, only a 12%‐14% 5‐year survival rate has been reached for patients with advanced‐stage CRC, which highlights the necessity to identify useful prognostic markers and to develop effective therapeutic strategies against CRC.^2^, ^3^

Human sirtuin 2 (SIRT2) belongs to the sirtuin family of NAD‐dependent protein deacetylases that have been found to be required for various physiological and pathological processes, including ageing‐associated metabolism, cell cycle regulation, DNA damage and repair and inflammation.^4^, ^5^, ^6^, ^7^ The functions of sirtuins are mainly achieved by their ability to interact with and deacetylate proteins, such as metabolic enzymes, transcription factors and histones that participate in different cellular processes.^8^, ^9^, ^10^, ^11^, ^12^ Recently, histone deacetylases have been widely demonstrated to be therapeutic targets in cancer.^13^, ^14^, ^15^, ^16^ Among these, SIRT2 was first identified as a tubulin deacetylase that prevents chromosomal instability by controlling mitotic checkpoint function during early metaphase.^17^ Notably, in human cancers, whether SIRT2 is an oncogene or a tumour suppressor are debated.^18^ SIRT2 deficiency promotes breast cancer tumorigenesis and hepatocellular carcinoma tumorigenesis in mice.^19^, ^20^, ^21^ On the other hand, SIRT2 works synergistically with HDAC6 in bladder cancer to promote cell invasion and migration.^22^ SIRT2 suppresses tumorigenesis and maintains genomic integrity by regulating APC/C activity, while the down‐regulation or inhibition of SIRT2 interferes with cell cycle progression, which promotes cell cycle arrest in vitro.^23^ In CRC, SIRT2 was reported to be involved in shikonin‐mediated CRC metastasis.^24^ However, the role and mechanism by which SIRT2 contributes to CRC development and progression, as well as the clinical significance of SIRT2 down‐regulation, are still unclear.

MicroRNAs (miRNAs) are small noncoding RNAs, and posttranscriptional gene expression is negatively regulated through miRNA base pairing with the 3′‐untranslated region (3′‐UTR) of specific target mRNAs.^25^, ^26^, ^27^ It has been demonstrated that miRNAs act on cell cycle progression, apoptosis, cell differentiation and several other key cellular processes.^25^, ^26^ Extensive studies have indicated that in cancers, miRNAs function as oncogenic miRNAs (oncomirs) and tumour suppressor miRNAs.^26^ In cancer cells, oncomirs are up‐regulated and contribute to carcinogenesis by inhibiting tumour suppressor genes, while tumour suppressor miRNAs inhibit oncogene expression, which prevents cancer development.^28^ Many studies have found miR‐212‐5p to be required for certain cell biology functions and the development of multiple organs, as well as in the invasion, migration and growth of various cancers, including CRC^29^, ^30^, ^31^, ^32^, ^33^, ^34^; hence, it may function as a promising new target for cancer therapy. This study demonstrates that SIRT2 is directly targeted by miR‐212‐5p and significantly down‐regulated in CRC and that the loss of SIRT2 contributes to CRC progression. Our findings provide a promising therapeutic target and prognostic biomarker for CRC diagnosis and treatment.

## 2 MATERIALS AND METHODS

### 2.1 Immunohistochemistry (IHC)

Superchip, Inc, was the source of the tissue microarrays of paired samples of adjacent normal tissue and primary CRC tissue, which were used to prepare 4‐μm‐thick paraffin‐embedded sections. All patients diagnosed with CRC had their diagnosis histologically confirmed by biopsy. The tissue sections were processed for deparaffinization/rehydration and antigen retrieval and stained for SIRT2 expression using a primary antibody against human SIRT2 (Cell Signaling Technology). Then, a horseradish peroxidase (HRP)‐conjugated secondary antibody (Dako; Agilent Technologies, Inc) was incubated with the slides. Protein expression was visualized using a 3,3′‐diaminobenzidine chromogenic substrate kit (Dako; Agilent Technologies, Inc). Two independent pathologists blindly scored the immunohistochemistry (IHC) results using a 400× microscope. For IHC of the CRC tissue microarray, each section was scored as a whole. Staining intensity was quantified using the values 3, 2, 1 or 0, indicating strong staining, moderate staining, weak staining or no staining, respectively. The percentage of positive cells was graded as follows: 3, >50%; 2, 10%–50%; 1, < 10%; and 0, negative. The product of staining intensity and grade was calculated and used as the IHC score, which was described as either high expression (>4) or low expression (0‐4) for each sample.

### 2.2 Cell culture

The CRC cell lines SW480, SW948, HCT116 and SW620 and the human intestinal epithelial cell line HIEC were procured from the American Type Culture Collection and maintained in DMEM (HyClone, Fisher Scientific) supplemented with 10% foetal bovine serum, streptomycin (100 μg/mL) and penicillin (100 units/mL) in a 5% (v/v) CO_2_ humidified atmosphere at 37°C. Cellular morphology and short tandem repeat analyses were conducted to authenticate all cell lines using an AmpF/STR Identifier Kit (Applied Biosystems).

### 2.3 Expression vectors, transfection and transduction

The downstream 3′‐UTR and the open reading frame were cloned between the *HindIII* and *EcoRI* sites into a cytomegalovirus promoter‐containing pcDNA 3.1 vector (Invitrogen) to construct an expression vector encoding human SIRT2. Once amplification of the small interfering RNA sequences against SIRT2 (shSIRT2; Table S2) was completed, cloning into a GV115 lentiviral vector (GeneChem) was performed. The plasmids were transfected into the indicated cells using HiPerFect (Qiagen), as instructed by the manufacturer. For stable cell line generation, lentiviral vectors were used at a multiplicity of infection of 100:1 to infect SW480 and SW620 cells. Real‐time PCR at 72 hours after infection was used to confirm the efficiency of infection. Puromycin (2 µg/mL; Gibco, Thermo Fisher Scientific) was used to select stable clones.

### 2.4 Luciferase assay

A miR‐212‐5p inhibitor, a synthetic miR‐212‐5p mimic and a negative control oligonucleotide were purchased from RiboBio. Once amplified, the wild‐type (WT) 3′‐UTR fragment of SIRT2 containing putative binding sites A and B for miR‐212‐5p was cloned between the *XhoI* and *NotI* sites in a psiCHECK‐2 vector downstream of the SV40 promoter‐driven Renilla luciferase cassette (Promega). Respective mutations of miR‐212‐5p‐binding sites A and B were created using a site‐directed mutagenesis kit (Agilent Technologies), as instructed by the manufacturer. Twenty‐four hours prior, SW480 cells were seeded into 24‐well plates for co‐transfection with the target sequence (WT or mutant 3′‐UTR) expression vectors and miRNA (the miR‐212‐5p mimic, miR‐212‐5p inhibitor or negative control) with the aid of Lipofectamine 2000 (Invitrogen). After 48 hours, the Renilla and firefly luciferase activities were measured by means of a Dual‐Luciferase Reporter Assay System (Promega), as instructed by the manufacturer. Normalization of the results was performed using Renilla luciferase activity. Each experiment was performed in triplicate.

### 2.5 Colony formation assay

Cells at a density of 1 × 10^3^ cells per well were seeded into 6‐well plates and grown at 37°C for 10 days. Thereafter, the cells were fixed using methanol/acetone (1:1), and crystal violet (Yeyuan) was used to stain the cells. A GelCount colony counter (Oxford Optronix, Inc) was used to count colonies containing 50 cells, while an EVOS microscope (Advanced Microscopy Group) was used to capture images at 200× magnification.

### 2.6 Cell proliferation assay

Cells at a density of 2 × 10^5^ cells per well were seeded into 6‐well plates and grown overnight. Then, transfection of the cells with the indicated constructs or oligonucleotides was performed. Cells were harvested after 48 hours and seeded at a density of 3 × 10^3^ cells per well into 96‐well plates. Each well was incubated for 2 hours at 37°C after adding ten microlitres of cell counting kit‐8 (CCK‐8) solution (Beyotime). A multimode microplate reader (Thermo Fisher Scientific) was utilized to read the absorbance at 450 nm.

### 2.7 Flow cytometry

Cell apoptosis and cell cycle arrest were examined using flow cytometry analyses. Cells were seeded at a density of 2 × 10^5^ per well into 6‐well plates for cell cycle analysis. The cells were collected 48 hours after transfection, fixed with ice‐cold 70% ethanol and incubated for 24 hours at 4°C. Propidium iodide (PI, 50 µg/mL; BD Biosciences) was used to stain the cells. A FACSCanto cell sorter (BD Biosciences) was used for cell cycle analysis. After 48 hours of transfection, the cells were harvested to measure apoptosis. An Annexin V‐FITC Apoptosis Detection Kit (BD Biosciences) was utilized to examine the cells after resuspension in staining buffer. Annexin V‐positive/PI‐negative stained cells were considered apoptotic. The CellQuest Pro software (BD Biosciences) and flow cytometry (BD Biosciences) were utilized for data analysis.

### 2.8 Invasion and migration assays

In brief, at a density of 2 × 10^3^ cells/well, the upper chamber of 24‐well chambers was filled and cultured in serum‐free medium, while 10% FBS was added in the lower chamber. Forty‐eight hours later, 0.5% crystal violet was used to fix and stain the cells that had migrated. Then, cells in three randomly selected fields were counted under a microscope. Matrigel (500 ng/mL; BD Biosciences) was used to pre‐coat the seeded cells for the invasion assay, while the other steps were identical to those of the migration assay.

### 2.9 Quantitative real‐time PCR

Total RNA extraction from cells or freshly frozen CRC tissues was performed with the TRIzol Reagent (Invitrogen), and RNA was purified with a miRNeasy Kit (Qiagen). RNA quantity was determined by reading the absorption at 260 nm. Applied Biosystems was the source of the TaqMan miRNA Reverse Transcription Kit (used to transcribe 10 ng of total RNA into cDNA for miRNA detection). The TaqMan Fast Advanced Master Mix (Applied Biosystems) was utilized to conduct real‐time PCR in triplicate. A QuantiTect Reverse Transcription Kit (Qiagen) was used to synthesize cDNA from 1 µg of total RNA for mRNA detection. SYBR Premix Ex Taq (Takara Bio, Inc) was utilized to conduct real‐time PCR in triplicate. β‐Actin was used as the internal control for mRNA detection, while U6 small nuclear RNA (U6) was used as the internal control for miRNA detection. All PCR primer sequences (GenePharma) are shown in Table S2. RiboBio was the source of the U6 and PCR primers for miR‐212‐5p. The $2^{‐\Delta \Delta C_{t}}$ method was utilized for data analysis.

### 2.10 Western blot assay

Cell lysates were obtained on ice using lysis buffer (Beyotime), followed by centrifugation at 16 000 *g* for 10 minutes. Thereafter, the lysate was heated at 95°C for 5 minutes in loading buffer. Sodium dodecyl sulphate‐polyacrylamide gel electrophoresis (10%) was used to separate the proteins. After being transferred onto a nitrocellulose membrane (Merck Millipore), the separated proteins were blocked for 2 hours using 5% non‐fat milk or bovine serum albumin. Overnight incubation at 4°C was conducted after adding the primary antibody against SIRT2 (Cell Signaling Technology; dilution 1:1000) or β‐actin (Sigma‐Aldrich; dilution 1:2000). An HRP‐conjugated secondary antibody (Cell Signaling Technology; dilution 1:2000) was added to the membrane and incubated for 1 hours at room temperature. Then, the membrane was washed three times using Tris‐buffered saline containing Tween 20 (TBST). After incubation, additional rinses were performed using TBST. A Molecular Imager ChemiDoc XRS + Imaging System was used to scan the protein bands, and the Image Lab software (Bio‐Rad Laboratories) was used for quantification after enhanced chemiluminescence reagents (Thermo Fisher Scientific) were employed to detect chemiluminescence signals.

### 2.11 In vivo tumorigenicity assay

The Fourth Military Medical University's Use of Live Animals in Teaching and Research (CULATR) Committee provided approval for all procedures on animals. Following the animal care guidelines of the institution, BALB/c nude mice that were 6‐8 weeks of age were housed in a standard specific pathogen‐free environment. Female BALB/c nude mice were allocated into each group (n = 10), and each flank was subcutaneously injected with 10^7^ cells, as indicated, in 200 µL of PBS. Tumour diameter was measured every 3 days to determine the tumour growth rate. A slide calliper was used to measure the minimum (W) and maximum (L) length of the tumours, and the formula *V* = 1/2(L × W^2^) was used to calculate the tumour volume. Thirty days following injection, the mice were sacrificed and each tumour was isolated and weighed.

### 2.12 In vivo metastasis assay

In total, 1 × 10^6^ cancer cells were injected into the tail veins of 8‐week‐old female nude mice to create experimental lung metastasis models. For in vivo bioluminescence imaging, 10 minutes before imaging, the tumour‐bearing mice were anaesthetized using an intraperitoneal injection of 3 mg of D‐luciferin (VivoGlo Luciferin; Promega). A Xenogen IVIS system (Xenogen Corporation) was used every week after injection to monitor luciferase‐expressing tumour cell bioluminescence. Histological examinations for lung metastases were carried out upon death or after sacrificing the mice 8 weeks after the initial injections.

### 2.13 Online databases

The miRWalk 2.0 (http://zmf.umm.uni‐heidelberg.de/apps/zmf/mirwalk2/) and TargetScanHuman 7.2 (http://www.targetscan.org/vert_72/) databases were used to analyse the binding site interactions between genes and miRNAs.

### 2.14 Statistical analysis

All experiments were performed in triplicate. The SPSS software v.19.0 (SPSS, Inc) was used for statistical analysis. A mean ± SEM format is used for data presentation. Student's unpaired *t* test was utilized for comparisons between groups. Differences between groups were evaluated by one‐way analysis of variance, with the least significant difference or Dunnett's post hoc test for post hoc analysis. The log‐rank test was utilized to identify significant differences between subgroups, while overall survival curve analysis used the Kaplan‐Meier method. The chi‐square test was utilized to compare differences between categorical variables. The correlation between SIRT2 expression and miR‐212‐5p was assessed using the Spearman rank test. The associations between variables and patient survival time were analysed using the multivariate Cox proportional hazards method. A *P* value <.05 was assumed to indicate statistical significance.

## 3 RESULTS

### 3.1 SIRT2 is significantly down‐regulated in CRC cells and tissues

**FIGURE 1:**
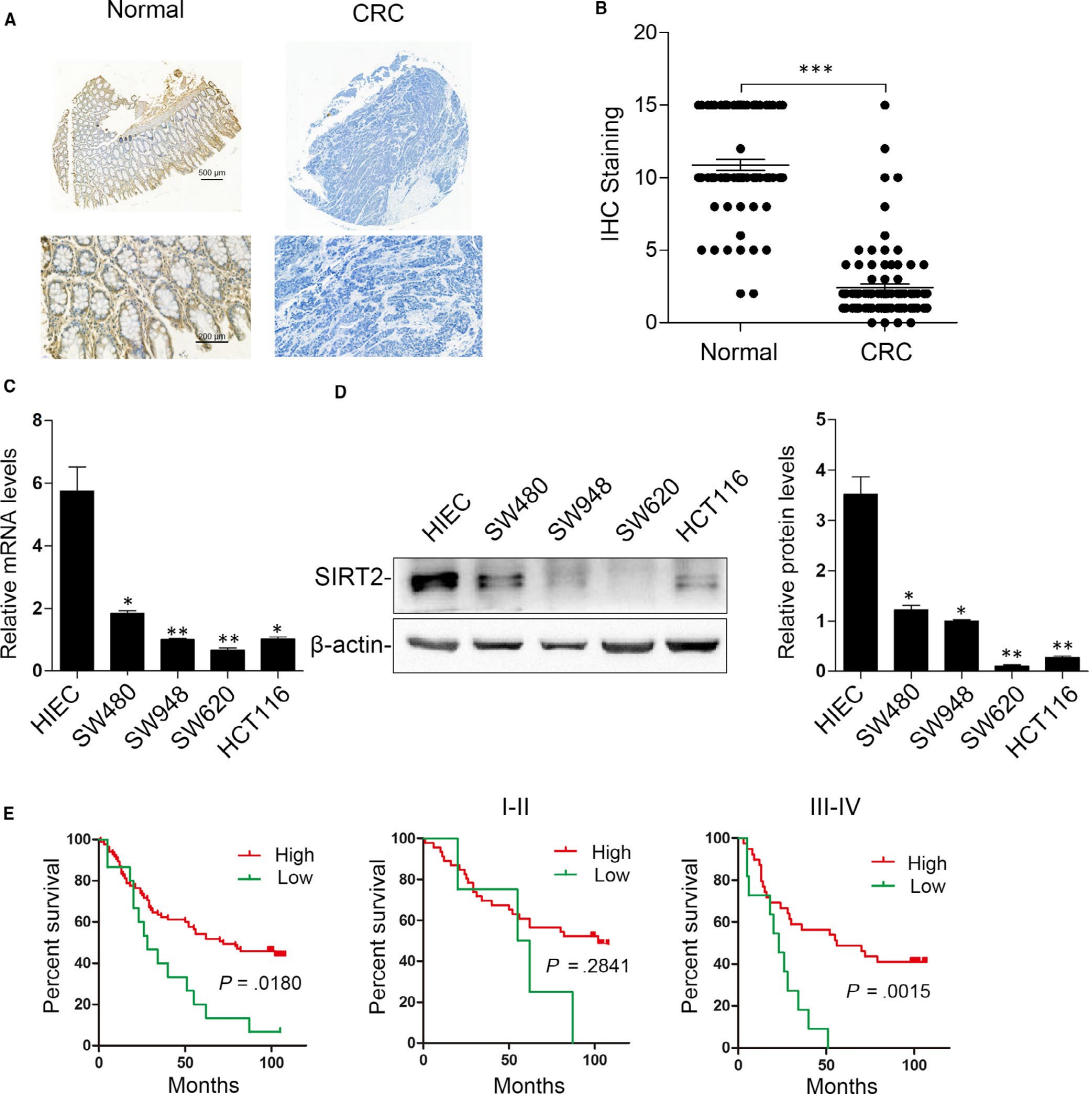
SIRT2 is down‐regulated in colorectal cancer (CRC) tissues, and the loss of SIRT2 is correlated with a poor prognosis in patients with CRC. A, Representative images of IHC staining of SIRT2 expression. Samples were obtained from 100 pairs of normal colorectal tissues (left) and primary CRC tissues (right). B, Quantification of IHC staining. ***P < .001. C, SIRT2 mRNA levels in HIEC and CRC cell lines were detected by real‐time PCR. β‐actin was used as an internal control. **P < .01, *P < .05; n = 3. D, Western blot assay for SIRT2 protein expression in HIEC and CRC cell lines. β‐Actin was used as an internal control (n = 3). E, Kaplan‐Meier analysis of survival time in all patients with CRC (n = 100), stage I–II patients (n = 40) and stage III‐IV patients (n = 60). The log‐rank test was used to calculate *P* values

The SIRT2 expression pattern was analysed in tissue microarrays containing 100 pairs of CRC and adjacent healthy colorectal epithelial tissues through IHC staining to investigate the role of SIRT2 in CRC. SIRT2 was significantly down‐regulated in CRC tissues relative to adjacent normal tissues (Figure 1A,B). SIRT2 expression was primarily localized in the cytoplasm (Figure 1A). In addition, as shown in Figure 1C,D, both the protein and mRNA expression levels of SIRT2 were found to be decreased in CRC cell lines compared with the healthy epithelial cell line HIEC. These data imply that the loss of SIRT2 expression may be involved in CRC development.

### 3.2 Correlation between clinicopathological characteristics and SIRT2 expression in CRC patients

We next sought to determine whether there is a correlation between SIRT2 expression and clinicopathological characteristics in patients with CRC. As shown in Table 1, the absence of SIRT2 was correlated with the American Joint Committee on Cancer (AJCC) stage, distant metastasis and lymph node metastasis in CRC (Table 1). Furthermore, SIRT2 down‐regulation was shown to be an independent prognostic factor for CRC through multivariate Cox regression analysis (Table 2).

**TABLE 1:** Correlation between SIRT2 expression and the clinicopathological characteristics of CRCs in a cohort of human CRC tissues (n = 100)

Clinicopathological variables	Tumour SIRT2 expression		*P* value
	Low (n = 67)	High (n = 33)	
Age (years)			
≤60	27	15	.620
>60	40	18	
Sex			
Female	33	17	.851
Male	34	16	
Tumour location			
Right colon	34	13	.209
Left colon	25	11	
Rectum	8	9	
Tumour size			
≤5 cm	31	15	.834
>5 cm	36	18	
Tumour differentiation			
Good or moderate	32	27	.002
Poor	35	6	
Tumour invasion			
T1	10	12	.013
T2	14	9	
T3	22	8	
T4	21	4	
Lymph node metastasis			
Absent	20	23	<.001
Present	47	10	
Distant metastasis			
Absent	45	29	.038
Present	22	4	
AJCC stage			
Stage I	8	8	.032
Stage II	12	12	
Stage III	24	10	
Stage IV	23	3	

Abbreviations: AJCC, American Joint Committee on Cancer.

**TABLE 2:** Univariate and multivariate analysis of factors associated with survival in human CRCs (n = 100)

Variables	Univariate analysis			Multivariate analysis		
	HR	95% CI	*P* value	HR	95% CI	*P* value
Age	0.976	0.965‐1.010	.712			
Sex (female vs male)	1.124	0.902‐1.503	.181			
Tumour size (≤5 vs > 5 cm)	0.801	0.603‐1.081	.164			
Tumour differentiation (well/moderate vs poor)	0.183	0.134‐0.245	<.001	0.776	0.524‐1.103	.253
Tumour invasion (T1‐T3 vs T4)	0.362	0.233‐0.461	<.001	0.654	0.203‐2.063	.442
Lymph node metastasis (absent vs present)	0.125	0.095‐0.192	<.001	0.315	0.108‐0.176	.044
Distant metastasis (absent vs present)	0.105	0.073‐0.142	<.001	0.463	0.309‐0.741	.001
AJCC stage (I‐II vs III‐Ⅳ)	0.129	0.091‐0.179	<.001	0.481	0.339‐0.684	<.001
SIRT2 expression (high vs low)	0.218	0.169‐0.326	<.001	0.403	0.293‐0.563	<.001^a^

Abbreviations: AJCC, American Joint Committee on Cancer.

^a^ SIRT2 down‐regulation was shown to be an independent prognostic factor for CRC.

### 3.3 Loss of SIRT2 indicates a poor prognosis of CRC

The log‐rank test and Kaplan‐Meier analysis were used to clarify the association between CRC patient survival time and SIRT2 expression to investigate the potential prognostic value of SIRT2 levels in CRC. As shown in Figure 1E, the loss of SIRT2 expression was significantly correlated with decreased survival time in patients with CRC. Interestingly, we found that the absence of SIRT2 was correlated only with advanced CRC (stages IV and III) but not early CRC (stages II and I). These results indicate that the decreased expression of SIRT2 is helpful for CRC prognosis prediction during advanced stages.

### 3.4 CRC cell proliferation, invasion and migration are inhibited by SIRT2 overexpression

**FIGURE 2:**
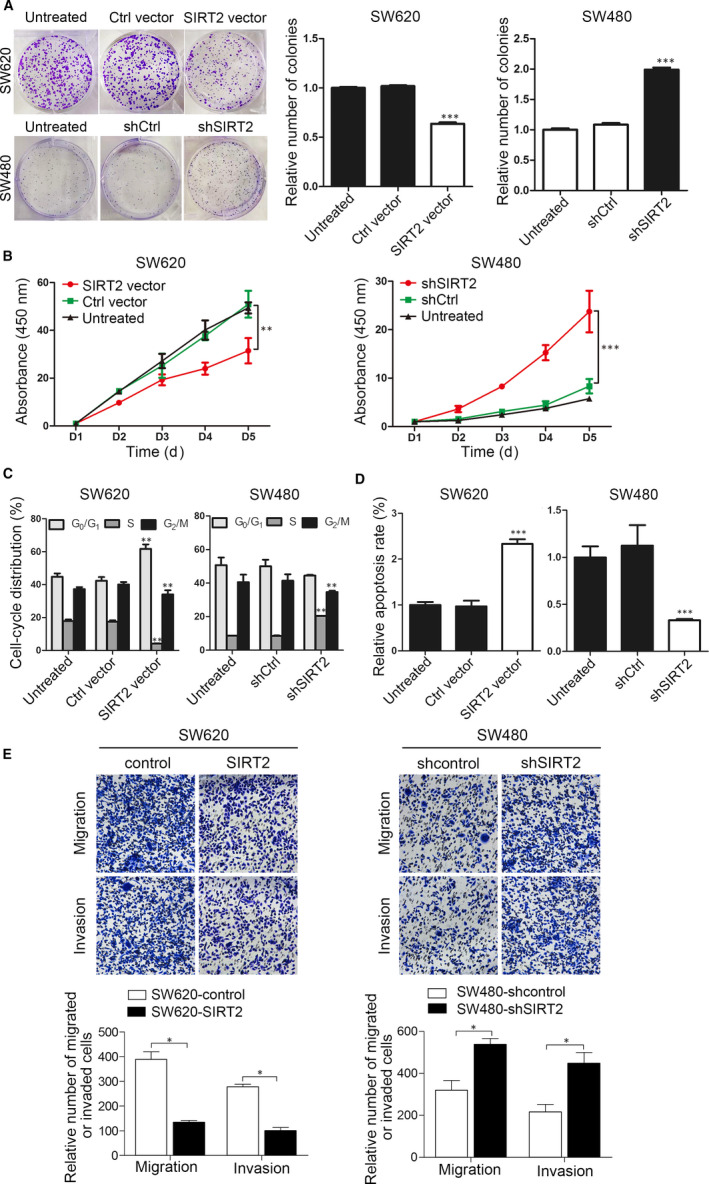
SIRT2 inhibits the proliferation, migration and invasion of CRC cells. SW480 and SW620 cells were transfected with SIRT2‐ and shSIRT2‐expressing vectors, respectively. A, Colony formation was measured 10 d after transfection. ***P < .001 vs the untreated group; n = 3. B, Cell proliferation was measured at the indicated time points using the CCK‐8 assay. **P < .01, ***P < .001 vs the untreated group; n = 3. C, D, The cells were stained with PI or annexin V‐FITC/PI. Cell cycle (C) and apoptosis (D) were assessed using flow cytometry. E, Transwell assay analysis of the migration and invasion of the indicated CRC cells. *P < .05, **P < .01, ***P < .001 vs the untreated group; n = 3

To further explore the function of SIRT2 in human CRC cells, we used vectors expressing SIRT2 or shSIRT2 to overexpress or knock down SIRT2, respectively, in SW620 and SW480 CRC cells (Figure S1A‐D). Relative to control cells (untreated and empty vector‐transfected cells), colony formation was significantly inhibited in SIRT2‐overexpressing SW620 cells and significantly promoted in SIRT2‐knockdown SW480 cells (Figure 2A). Consistent with these results, SIRT2 overexpression suppressed the proliferation of SW620 cells, while SIRT2 knockdown enhanced the proliferation of SW480 cells (Figure 2B). Flow cytometry assays showed that SIRT2 overexpression induced cell cycle arrest in G_0_/G_1_ phase and significant apoptosis in SW620 cells, while the opposite effects on cell cycle progression and apoptosis were observed in SIRT2‐deficient SW480 cells (Figure 2C,D). In addition, Transwell assays showed that compared with the SW620‐control group, the number of tumour cells migrating or invading out of the chamber after SIRT2 overexpression was significantly lower. In contrast, SIRT2 down‐regulation in SW480 cells significantly enhanced the number of migrating or invading tumour cells compared with the number of migrating and invading control cells (Figure 2E). Moreover, using Western blotting analysis, we found that SIRT2 overexpression suppressed the levels of cyclin D1, MMP2 and MMP9, while SIRT2 knockdown induced the expression of cyclin D1, MMP2 and MMP9 (Figure S2). Overall, these results demonstrate that SIRT2 inhibits CRC cell proliferation, invasion and migration.

### 3.5 SIRT2 suppresses the growth and metastasis of CRC in vivo

**FIGURE 3:**
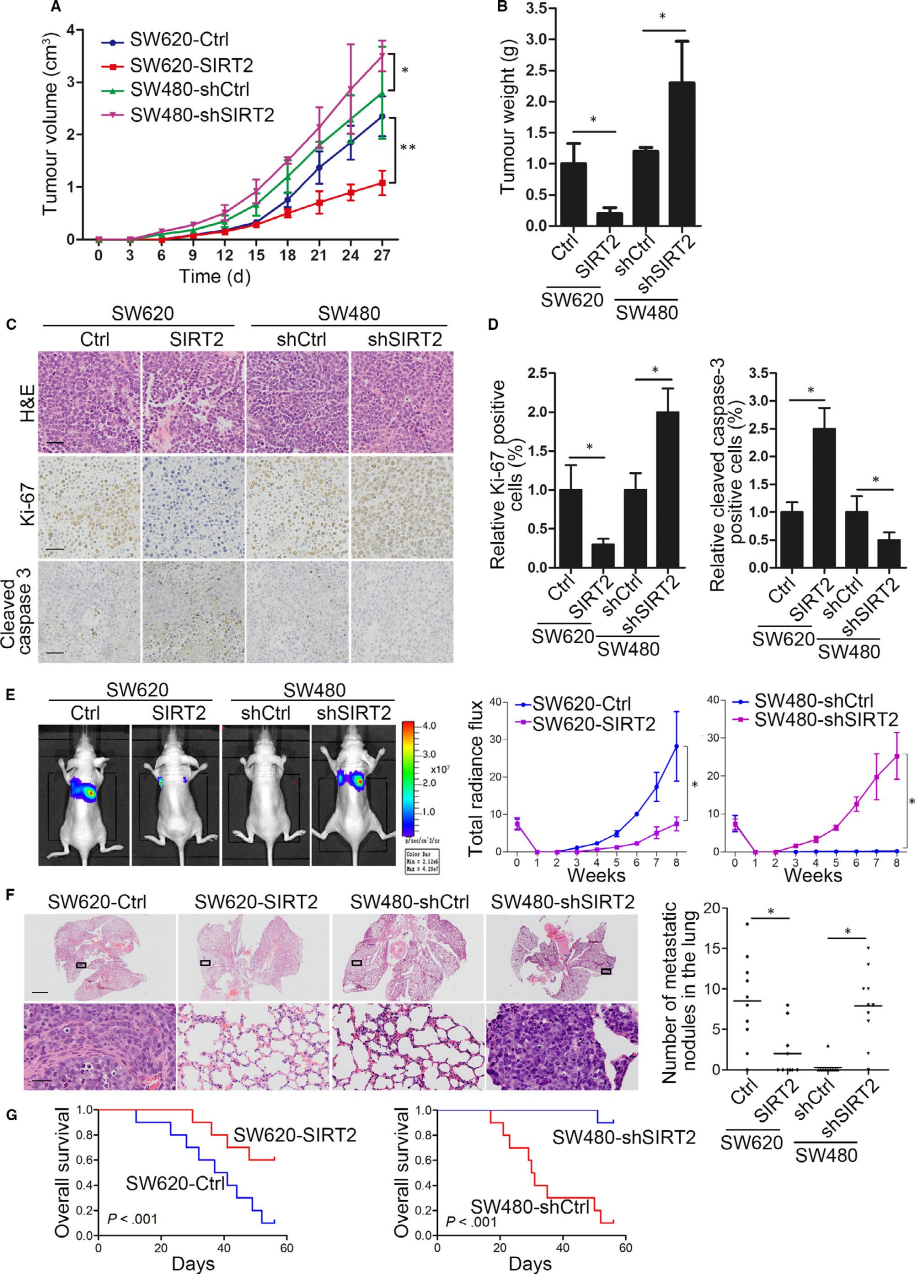
SIRT2 suppresses CRC cell tumorigenesis and metastasis in vivo. A, B Quantification of tumour weights (A) and tumour growth curves (B) in mice. C, Representative images of tumour samples that were stained with haematoxylin and eosin (H&E), Ki67 and cleaved caspase 3 by IHC staining. D, The percentages of Ki67‐ and cleaved caspase 3‐positive cells were measured. Bars: (main) 100 µm; (insets) 25 µm. E, The indicated cell lines were transplanted into the tail veins of nude mice. Left, representative bioluminescent imaging of the different groups 8 wk after implantation. Right, the total flux curve for each group. F, Left, representative H&E staining of lungs from each group. Number of lung metastatic foci in the different groups. G, Overall survival of nude mice in the different groups. Error bars show the SD. *P < .05; **P < .01

To determine the functionality of SIRT2 in CRC in vivo, the growth and metastasis of CRC cell xenografts in nude mice were assessed. For tumorigenesis assays, the subcutaneous injection of cells that stably expressed SIRT2, shSIRT2 or their controls into the flanks of nude mice was followed by monitoring tumour volumes. In vivo, we found that SIRT2 overexpression significantly suppressed tumour growth, whereas SIRT2 inhibition promoted tumour growth (Figure 3A–C). Ki67 and cleaved caspase 3 levels were determined in the tumours using IHC staining. SW620‐SIRT2 mice with SIRT2 overexpression showed a significantly decreased percentage of Ki67‐positive cells and increased cleaved caspase 3 staining compared with negative control mice. In contrast, the percentages of Ki67‐positive cells increased after SIRT2 inhibition, while those of cleaved caspase 3‐positive cells decreased in SW480‐shSIRT2 mice (Figure 3D).

In addition, in the in vivo metastasis assays where cells were administered into nude mice through tail vein injection, we found that the enforced expression of SIRT2 prominently decreased the bioluminescence signals in SW620‐SIRT2 tumour‐bearing mice compared with those in SW620‐control mice (Figure 3E). In contrast, SW480‐shSIRT2 tumour‐bearing mice displayed a higher signal intensity than SW480‐shcontrol mice (Figure 3E). Histological analysis indicated that SIRT2 overexpression significantly decreased the number of lung metastatic nodules in SW620‐SIRT2 cells. SIRT2 knockdown enhanced lung metastasis in vivo in SW480‐shSIRT2 cells compared with SW480‐shcontrol cells (Figure 3F). SIRT2 up‐regulation also increased the overall survival time of the SW620‐SIRT2 group, while decreased overall survival times were observed in the SW480‐shSIRT2 group (Figure 3G). Taken together, these in vivo findings confirm that SIRT2 acts as a tumour suppressor of CRC cell metastasis and growth.

### 3.6 SIRT2 is directly targeted by miR‐212‐5p

**FIGURE 4:**
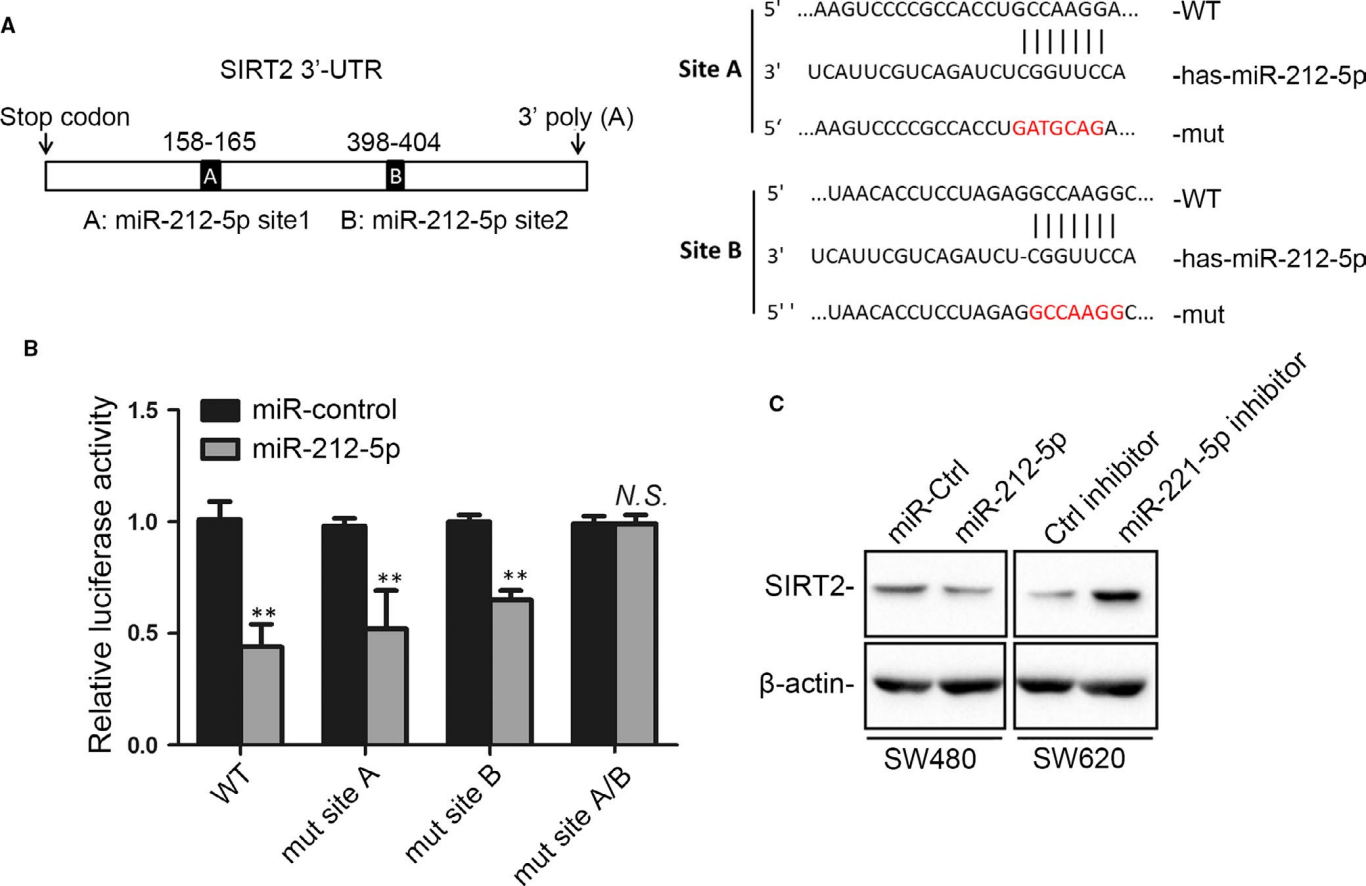
miR‐212‐5p down‐regulates SIRT2 by targeting the 3′‐UTR of SIRT2 mRNA. A, Schematic diagram of the SIRT2 3′‐UTR with two putative miR‐212‐5p binding sites, A and B (left), and WT and site A‐ and site B‐mutated SIRT2 3′‐UTRs (right). B, SW480 cells were co‐transfected with miR‐212‐5p and promoter‐driven luciferase reporter constructs expressing WT or mutated SIRT2 3′‐UTR. Luciferase activity was measured. **P < .01 vs the control group; n = 3. N. S., not significant. C, Western blot assay for SIRT2 expression in SW480 and SW620 cells transfected with miR‐212‐5p and anti‐miR‐212‐5p, respectively. β‐actin was used as an internal control. UTR, untranslated region; WT, wild‐type; mut, mutant

To investigate the possible mechanism that leads to SIRT2 down‐regulation in CRC tissues, we predicted the potential miRNA target sites in SIRT2 mRNA using the bioinformatic databases TargetScanHuman 7.2 and miRWalk 2.0. As a result, miR‐212‐5p, the only miRNA that is broadly conserved in the SIRT2 mRNA 3′‐UTR binding site, drew great attention. Further sequence analysis indicated the presence of two putative binding sites (sites A and B) for miR‐212‐5p in the SIRT2 3′‐UTR. The transient transfection of miR‐212‐5p, a WT control and site A‐ and/or site B‐mutated SIRT2 3′‐UTR into SW620 cells was performed to examine whether miR‐212‐5p regulates SIRT2 with luciferase assays. As shown in Figure 4B, the luciferase activities of the WT control and site A‐ and site B‐mutated SIRT2 3′‐UTRs, but not the double site A‐ and site B‐mutated SIRT2 3′‐UTR, were all markedly decreased by miR‐212‐5p. These results indicate that miR‐212‐5p may regulate SIRT2 by targeting both sites A and B in the SIRT2 3′‐UTR. To further validate SIRT2 regulation by miR‐212‐5p, Western blotting was performed on anti‐miR‐212‐5p‐ and miR‐212‐5p mimic‐transfected SW620 and SW480 cells, respectively. Figure 4C shows the down‐regulation of SIRT2 expression by the miR‐212‐5p mimic and the up‐regulation of SIRT2 by anti‐miR‐212‐5p, suggesting that in CRC cells, SIRT2 is directly targeted by miR‐212‐5p.

### 3.7 SIRT2 is important for miR‐212‐5p‐mediated CRC progression

**FIGURE 5:**
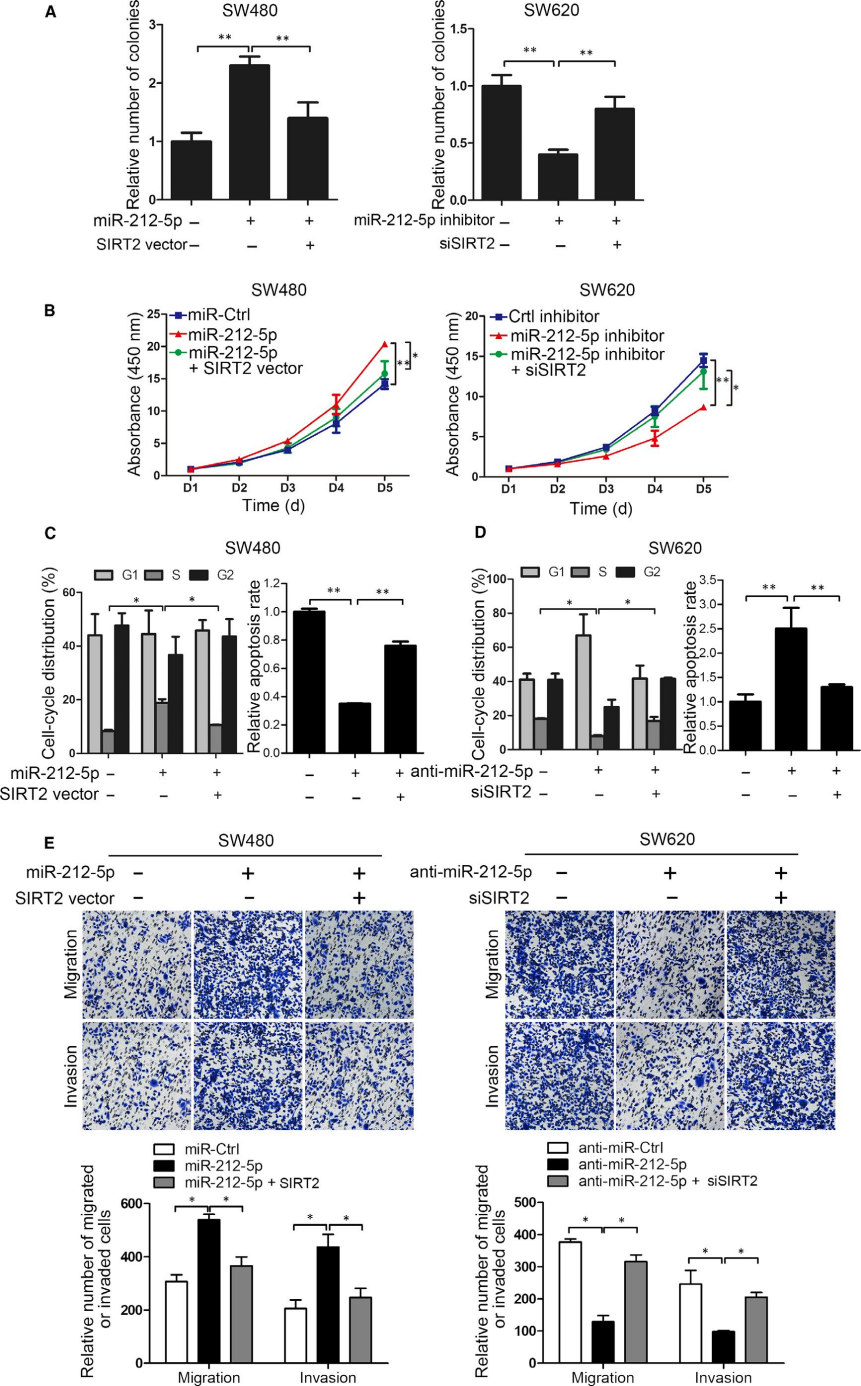
SIRT2 is a functional target of miR‐212‐5p. A, Colony formation, (B) proliferation, (C) cell cycle, (D) apoptosis and (E) Transwell migration and invasion were analysed after the transfection of SW620 cells with miR‐212‐5p alone and miR‐212‐5p plus the SIRT2‐expressing vector as well as after the transfection of SW480 cells with anti‐miR‐212‐5p alone and anti‐miR‐212‐5p plus the siSIRT2‐expressing vector. *P < .05, **P < .01; n = 3

Next, we performed rescue experiments to determine whether SIRT2 is involved in the miR‐212‐5p‐mediated regulation of CRC progression. SIRT2 overexpression reversed the promoting effect of miR‐212‐5p on SW620 colony formation and the proliferation rate, while SIRT2 knockdown restored the suppressive effects of anti‐miR‐212‐5p on SW480 colony formation and cell proliferation (Figure 5A,B). Similar effects were observed when SIRT2 overexpression resulted in miR‐212‐5p mimic‐mediated CRC cell cycle arrest and apoptosis, while SIRT2 down‐regulation restored these effects of miR‐212‐5p inhibitors (Figure 5C,D). Additionally, Transwell assays showed that SIRT2 up‐regulation significantly decreased the miR‐212‐5p‐enhanced migratory and invasive abilities, whereas SIRT2 down‐regulation rescued these effects (Figure 5E). These data show that SIRT2 is important for miR‐212‐5p‐mediated CRC progression as a result of its function as a downstream target gene of miR‐212‐5p.

### 3.8 SIRT2 levels are negatively correlated with miR‐212‐5p expression

**FIGURE 6:**
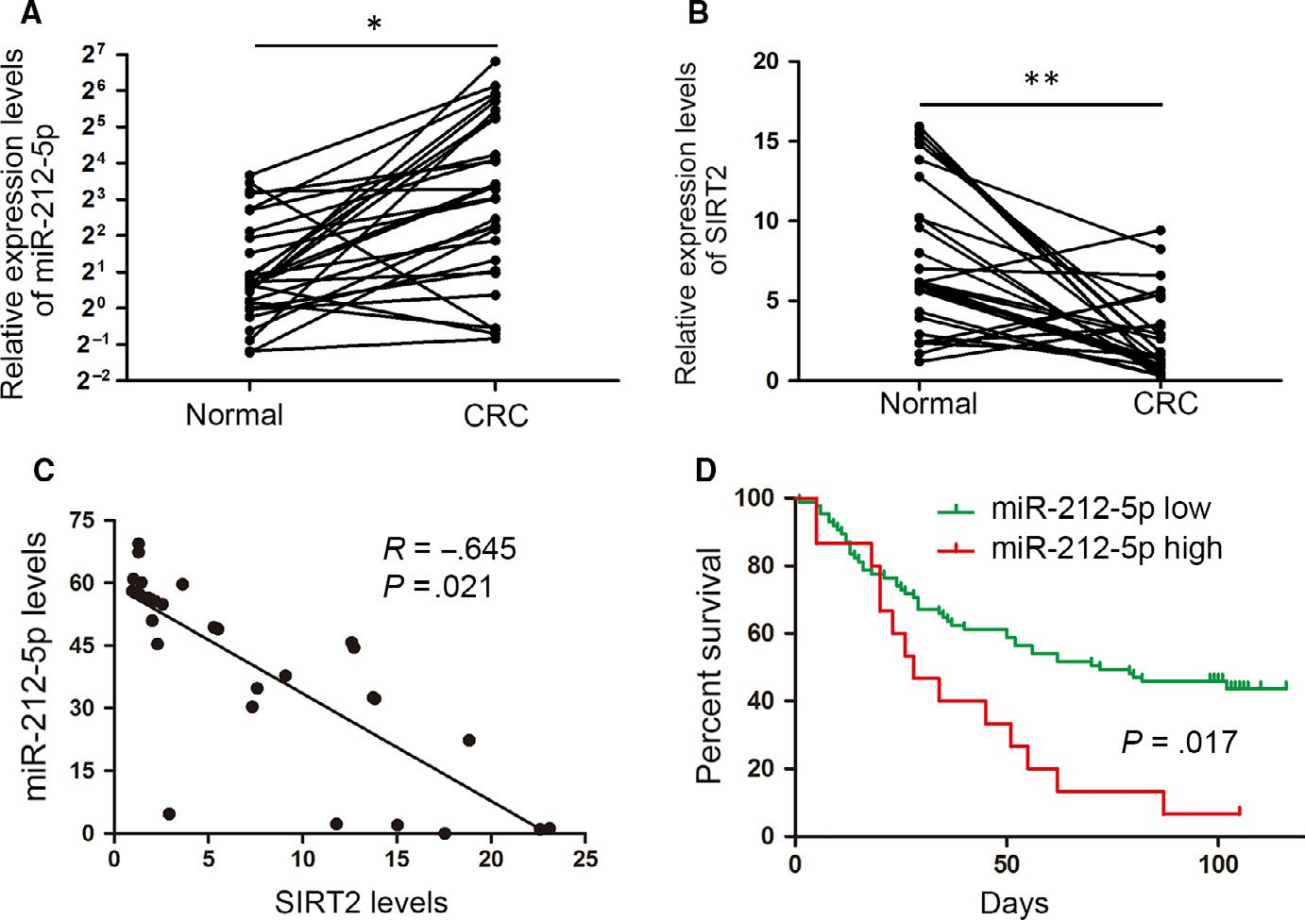
miR‐212‐5p and SIRT2 levels are clinically correlated. The expression of (A) miR‐212‐5p and (B) SIRT2 in paired CRC tumour tissue and adjacent normal colorectal tissue (n = 20) samples was evaluated by real‐time PCR. C, The correlation of miR‐212‐5p and SIRT2 expression was assessed by the Spearman rank test. D, Kaplan‐Meier analysis of survival time in all CRC patients with differential miR‐212‐5p expression

The expression of miR‐212‐5p was detected in paired primary CRC and adjacent normal tissues to further explore the clinical significance of miR‐212‐5p in CRC. The results demonstrated the up‐regulation of miR‐212‐5p in CRC tumour tissues relative to normal tissues, which is in contrast to the expression of SIRT2 (Figure 6A,B). Further correlation analysis suggested that miR‐212‐5p expression was inversely correlated with SIRT2 levels (Figure 6C). We next analysed the relationship between clinicopathological characteristics and miR‐212‐5p expression in CRC. In contrast to SIRT2, a positive correlation was found between miR‐212‐5p up‐regulation and tumour differentiation, metastasis and the AJCC stage (Table S1). An association between the high expression of miR‐212‐5p and the shortened survival time in patients with CRC was found using Kaplan‐Meier analysis (Figure 6D). Collectively, these results demonstrate the presence of an inverse correlation between miR‐212‐5p and SIRT2 expression, as well as its correlation with a poor prognosis in CRC.

## 4 DISCUSSION

SIRT2 functions as an NAD^+^‐dependent lysine deacetylase and belongs to the class III histone deacetylases (HDACs).^35^ SIRT2 is reported to be important for TNF‐alpha‐stimulated programmed necrosis in the setting of ischaemia‐reperfusion injury in the heart.^36^ In synchronized cells, SIRT2 delays mitotic progression by accumulating during mitosis, particularly at the G0/G1 transition checkpoint.^37^ In many human cancers, SIRT2 has been recognized as a tumour suppressor, and an association between mammary tumours, hepatocellular carcinomas and a loss of SIRT2 has been found in mice.^23^ SIRT2 sensitizes breast cancer cells to oxidative stress‐inducing agents by deacetylating and inhibiting the peroxidase activity of peroxiredoxin‐1.^19^ In non–small‐cell lung cancer cells, SIRT2 inhibits tumour growth by impairing Skp2‐mediated p27 degradation.^38^ Consistent with these findings, in this study, we found that compared with paired adjacent non‐cancerous tissue samples, SIRT2 was down‐regulated in CRC tissue samples. The loss of SIRT2 was correlated with aggressive clinicopathological features and indicated a poor CRC prognosis. Both in vitro and in vivo, SIRT2 overexpression was found to significantly inhibit the metastasis and proliferation of CRC, while SIRT2 down‐regulation exerted the opposite effect. However, there are examples where SIRT2 acts as an oncogene and may target various cancers. It has been reported that the inhibition of SIRT2 induces the apoptosis of HeLa cells and in turn regulates the down‐regulation of MDM2 and p300, leading to p53 accumulation.^39^ A SIRT2‐selective inhibitor was found to promote c‐Myc oncoprotein degradation and exhibit broad anticancer activity.^40^ These functions beyond cell context‐dependent functionality may be attributed to the fact that SIRT2 interacts with and deacetylates diverse target proteins participating in different cellular processes. Of note, the downstream substrates of SIRT2 in CRC remain to be explored, which will be our topic for further research.

Moreover, the regulatory mechanism of SIRT2 down‐regulation in human CRC remains unknown. miRNAs are a class of small noncoding RNAs with a length of 19‐22 nt that are involved in gene expression regulation at the posttranscriptional level through the degradation of specific target mRNAs and/or through the inhibition of their translation.^26^ They have been shown to act on several key cellular processes, such as apoptosis, cell cycle progression and cell differentiation.^25^ Extensive studies have revealed increasingly important roles for miRNAs in human cancer development and progression, and these roles are now believed to be some of the most prominent mechanisms in oncogene or tumour suppressor regulation.^26^ In this study, we predicted the possible miRNAs that target SIRT2 mRNA using the TargetScanHuman 7.2 and miRWalk 2.0 databases. Then, we verified that miR‐212‐5p can directly bind to the 3′‐UTR of SIRT2 mRNA, inhibiting its expression post‐transcriptionally. More importantly, our rescue experiments indicated that SIRT2 was important for miR‐212‐5p‐mediated CRC cell migration, invasion and proliferation. SIRT2 overexpression was found to restore the tumour‐suppressive effects of miR‐212‐5p up‐regulation. In clinical CRC tissues, miR‐212‐5p was markedly up‐regulated in CRC tumour tissues, which was consistent with the results of a previous study.^33^ miR‐212‐5p expression was inversely correlated with the levels of SIRT2. Collectively, these expression and functional results illustrate that miR‐212‐5p is involved in SIRT2 regulation during CRC progression. In addition to miR‐212‐5p, miR‐200c‐5p has also been found to specifically target SIRT2 in human pluripotent stem cell fate decisions.^41^ These findings indicate the precise spatiotemporal regulation of SIRT2 by miRNAs in different cellular contexts.

Particularly, we noticed that the study by Zhang et al had already revealed that SIRT2 is down‐regulated in CRC and that overexpression of SIRT2 inhibited CRC cell proliferation, migration and invasion, which validates the reliability of our study. However, there are findings that differ between our and Zhang et al's studies. First, they tested the biological role of SIRT2 on CRC cell proliferation, migration and invasion in vitro as well as on tumour growth in a nude mouse model, without examining the effect of SIRT2 in CRC metastasis in vivo. In our study, we established in vivo metastasis mouse models though tail vein injection of CRC cells. We found that the enforced expression of SIRT2 prominently suppressed SW620‐SIRT2 tumour metastasis compared with the SW620‐control group, while SW480‐shSIRT2 tumour‐bearing mice displayed increased metastasis in vivo, as evidenced by the bioluminescence signals, histological staining and survival analysis (Figure 3E,F). Second, in Zhang et al's study, the author revealed a potential regulatory mechanism for SIRT2 via phospho‐ERK inhibition in response to the antitumor compound Shikonin. However, it is unclear whether phospho‐ERK directly interacts with SIRT2 and whether phospho‐ERK inhibition increased SIRT2 protein levels. In our study, we found that SIRT2 is directly targeted by miR‐212‐5p. Using bioinformatics analysis and luciferase report assays, miR‐212‐5p was determined to directly bind to the 3′‐UTR of SIRT2 mRNA. Through further functional rescue experiments and clinical sample correlation analysis, we found that SIRT2 was functionally important for miR‐212‐5p‐mediated CRC progression, and the levels of SIRT2 are negatively correlated with miR‐212‐5p expression in clinical CRC tissues. These observations together provided strong evidence for the direct epigenetic regulation of SIRT2 loss in CRC. Third, we examined the expression of SIRT2 via immunohistochemical stain in the CRC tissue microarray with follow‐up data. As shown in Figure 1E, the loss of SIRT2 expression was significantly correlated with decreased survival time in patients with CRC. We further found that the absence of SIRT2 was only correlated with advanced CRC (stages IV and III), not early CRC (stages II and I). These results indicate that the loss of SIRT2 indicates a poor prognosis of CRC, which will be a great addition to Zhang et al's study. Taken together, our study revealed the novel findings that tumour‐suppressive SIRT2 is a direct functional target gene of miR‐212‐5p and that the miR‐212‐5p/SIRT2 axis is a promising prognostic factor and potential therapeutic target in CRC.

In summary, our study demonstrates that SIRT2 plays an important tumour‐suppressive role in CRC progression and serves as a promising diagnostic or prognostic biomarker, clinical predictor and therapeutic target for CRC diagnosis and treatment. We also highlight that miR‐212‐5p directly targets the SIRT2 gene, which is important for CRC progression mediated by miR‐212‐5p. The results of this study identify the miR‐212‐5p/SIRT2 axis as a new factor governing malignant cancer cell behaviours and clinical outcomes.

## CONFLICT OF INTEREST

No potential conflicts of interest were disclosed.

## AUTHOR CONTRIBUTIONS

**Feng Du:** Conceptualization (equal); Data curation (equal); Formal analysis (equal). **Zhijun Li:** Data curation (equal); Methodology (equal). **Guohua Zhang:** Resources (equal). **Shaoyan Si:** Investigation (equal); Methodology (equal). **Dejun Geng:** Conceptualization (equal); Project administration (equal). **Zhougen Tao:** Investigation (equal); Project administration (equal). **Kunhua Qiu:** Software (equal); Validation (equal). **Silei Liu:** Data curation (equal); Resources (equal). **Yu Zhou:** Investigation (equal); Validation (equal). **Yichao Zhang:** Validation (equal); Visualization (equal). **Jianwen Gu:** Conceptualization (equal); Project administration (equal); Supervision (equal). **Gang Wang:** Visualization (equal). **Lianyong Li:** Conceptualization (equal); Funding acquisition (equal); Project administration (equal); Supervision (equal). **Wei Wu:** Conceptualization (equal); Data curation (equal); Project administration (equal); Supervision (equal).

## Supporting information

Fig S1

Fig S2

Table S1

Table S2

## Data Availability

The data that support the findings of this study are available from the corresponding author upon reasonable request.
