# Supplementary figures and images for: SIRT2, a direct target of miR‐212‐5p, suppresses the proliferation and metastasis of colorectal cancer cells
Source: J Cell Mol Med. 2020 Jul 22;24(17):9985–98. doi: 10.1111/jcmm.15603 (PMC7520262; doi:10.1111/jcmm.15603)

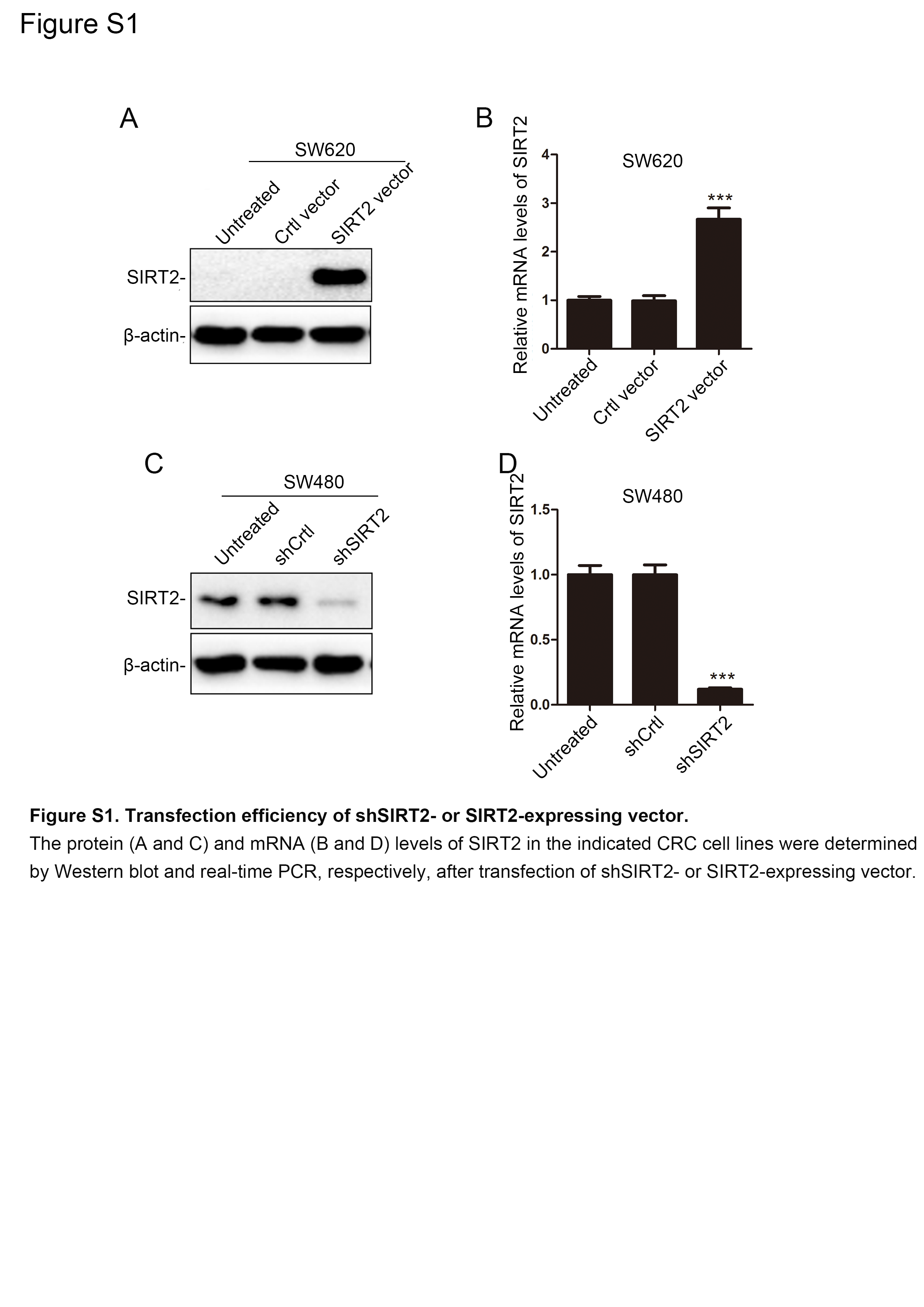

Supplement: Supplementary file 1 — Fig S1 [file JCMM-24-9985-s001.tif]

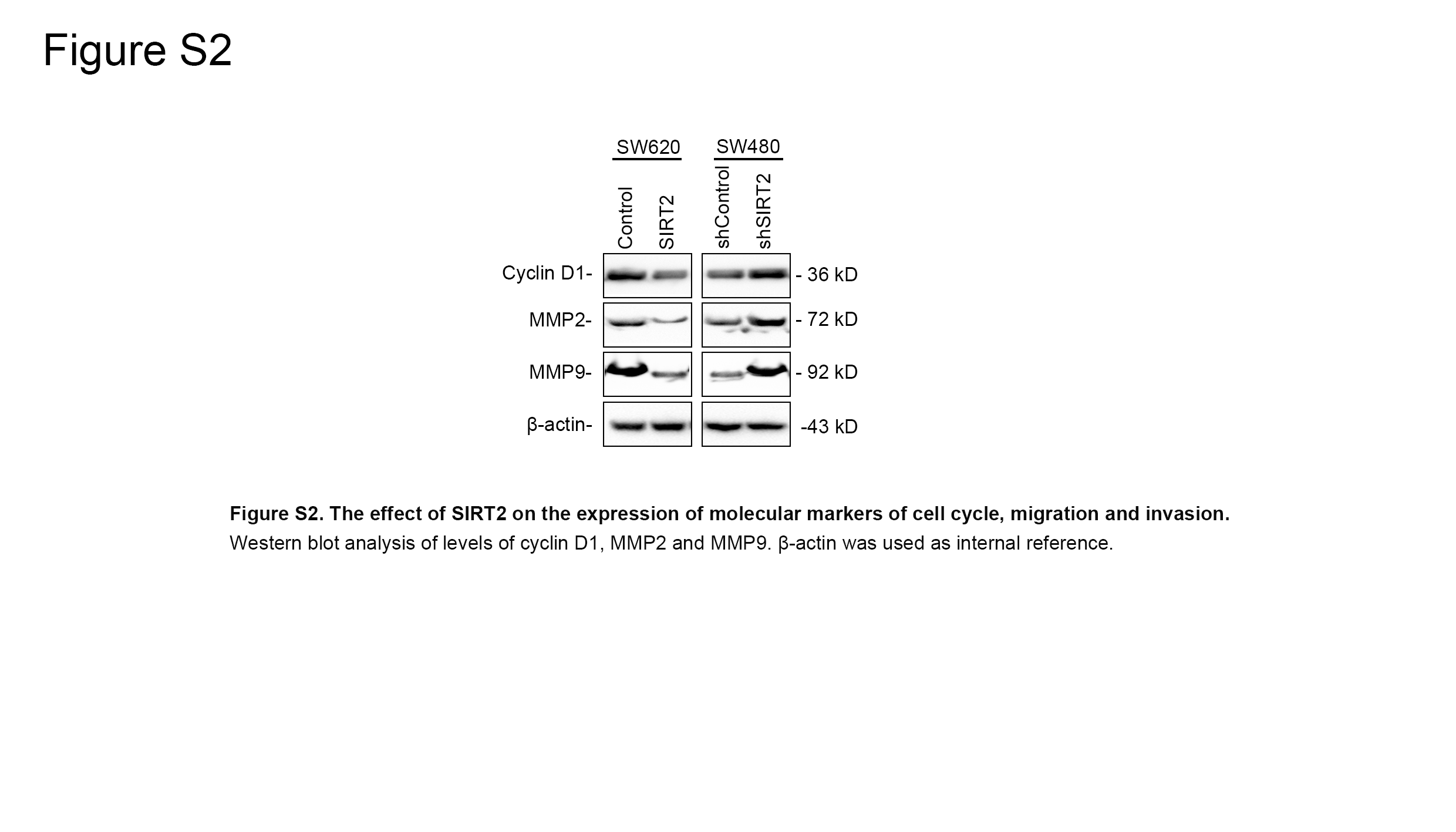

Supplement: Supplementary file 2 — Fig S2 [file JCMM-24-9985-s002.tif]
