# Supplementary material for: SIRT2, a direct target of miR‐212‐5p, suppresses the proliferation and metastasis of colorectal cancer cells
Source: J Cell Mol Med. 2020 Jul 22;24(17):9985–98. doi: 10.1111/jcmm.15603 (PMC7520262; doi:10.1111/jcmm.15603)
Supplement: Supplementary file 3 — Table S1 [file JCMM-24-9985-s003.docx]

Supplementary Table 1 Correlation between miR-212-5p expression and clinicopathological characteristics of CRCs in a cohort of human CRC tissues (n=100)

| **Clinicopathological variables** | | | Tumour miR-212-5p expression | |  |  |
| --- | --- | --- | --- | --- | --- | --- |
|  |  |  | Low  (n=42) | High  (n=58) |  |  |
| Age | ≤60 | | 17 | 28 | 0.367 |  |
|  | > 60 | | 25 | 30 |  |  |
| Sex | | female | 20 | 23 | 0.436 |  |
|  | | male | 22 | 35 |  |  |
| Tumour location | | right colon | 21 | 25 | 0.610 |  |
|  | | left colon | 14 | 20 |  |  |
|  | | rectum | 7 | 14 |  |  |
| Tumour size | | ≤5cm | 17 | 24 | 0.998 |  |
|  | | >5cm | 25 | 34 |  |  |
| Tumour differentiation | | well or moderate | 18 | 49 | <0.001 |  |
|  | | poor | 24 | 9 |  |  |
| Tumour invasion | | T1 | 1 | 1 | 0.416 |  |
|  | | T2 | 2 | 6 |  |  |
|  | | T3 | 25 | 38 |  |  |
|  | | T4 | 14 | 14 |  |  |
| Lymph node metastasis | | absent | 14 | 41 | <0.001 |  |
|  | | present | 28 | 17 |  |  |
| Distant metastasis | | absent | 26 | 55 | <0.001 |  |
|  | | present | 16 | 3 |  |  |
| AJCC* stage | | Stage I | 3 | 6 | <0.001 |  |
|  | | Stage II | 7 | 36 |  |  |
|  | | Stage III | 16 | 14 |  |  |
|  | | Stage IV | 19 | 9 |  |  |

*AJCC: American Joint Committee on Cancer
