# Supplementary material for: SIRT2, a direct target of miR‐212‐5p, suppresses the proliferation and metastasis of colorectal cancer cells
Source: J Cell Mol Med. 2020 Jul 22;24(17):9985–98. doi: 10.1111/jcmm.15603 (PMC7520262; doi:10.1111/jcmm.15603)
Supplement: Supplementary file 4 — Table S2 [file JCMM-24-9985-s004.docx]

Supplementary Table 2. Sequences for primers and RNAi used for indicated genes

| Gene name | Sequence (5’ to 3’) |
| --- | --- |
| ACTB-Forward primer | GGACTTCGAGCAAGAGATGG |
| ACTB-Reverse primer | AGCACTGTGTTGGCGTACAG |
| SIRT2-Forward primer | CACCTTCTACACATCACACTGCG |
| SIRT2-Reverse primer | TTCACACTTGGGCGTCACC |
| miR-212-5p Forward primer | GCTTACGCTTCGAGCCCAC |
| miR-212-5p Reverse primer | GACACCACGGCCCACTCTGCA |
| siSIRT2-1 | CGGCCTCTATGACAACCTA |
| siSIRT2-2 | CCGCTAAGCTGGATGAAAG |
| siSIRT2-3 | GCACCTTCTACACATCACA |
